# Adolescent and Young Adult Requests for Medication Abortion Through Online Telemedicine

**DOI:** 10.1001/jamahealthforum.2025.6808

**Published:** 2026-02-13

**Authors:** Dana M. Johnson, Jennifer E. Starling, Rebecca Gomperts

**Affiliations:** 1 University of Wisconsin-Madison School of Medicine and Public Health, Madison; 2 Mathematica Inc, Cambridge, Massachusetts; 3 Aid Access, Vienna, Austria

## Abstract

This cross-sectional study evaluates telehealth medication abortion rates among adolescents and young adults after the *Dobbs v Jackson Women’s Health Organization* decision by state-level restrictions and parental involvement laws.

## Introduction

Adolescents in the US experience many logistical and policy barriers to abortion care.^1,2^ Although most live in states with abortion restrictions,^3^ adolescents are often excluded from reproductive health research.^4^ To address this gap, we compared demand for online medication abortion among adolescents and young adults^5^ vs adults before and after the *Dobbs v Jackson Women’s Health Organization* decision, examining how demand varied across different state-level gestational bans and parental involvement laws.

## Methods

The University of Texas at Austin institutional review board approved this study. At the time of request, individuals consented to the use of anonymized data for research purposes. We followed the STROBE reporting guideline.

This cross-sectional study analyzed requests made to a high-volume online medication abortion telemedicine service, one of only a few services supporting all 50 states and Washington, DC, without any age restrictions (eMethods in Supplement 1).^6^ We compared request rates before (September 1, 2021-June 23, 2022) and after the *Dobbs* decision (June 24, 2022-October 31, 2023). For each period, we calculated the average weekly request rate per 100 000 female residents in 3 age groups: adolescents 15 to 17 years, young adults 18 to 24 years, and adults 25 to 49 years. We compared request rates across age groups, overall, and by type of state-level abortion restrictions in effect at the time of request. Rates were weighted by the population of female residents in each age group within the state ban type. Categories included overall; no ban or parental involvement law; no ban, parental involvement law; 12- to 24-week ban; 6-week ban; and total or near-total ban. For adolescents, we compared request rates across categories of parental involvement requirements at the time of request, including no parental involvement law, required parental notification, required parental consent, and required parental consent and notification.

We used 2-tailed *t* tests to examine differences, with statistical significance set at *P* < .05. We used R, version 4.5.1 (R Project for Statistical Computing) for analysis.

## Results

During the study period, the service received requests from all 50 states and Washington, DC, including 13 716 requests (6.0%) from adolescents, 97 768 requests (42.8%) from young adults, and 116 981 requests (51.2%) from adults. The mean weekly request rate per 100 000 female residents increased for all age groups baseline to post-*Dobbs:* for adolescents, 2.8 (95% CI, 2.5-3.0) to 8.6 (95% CI, 7.9-9.2), increasing 5.8 (95% CI, 5.1-6.5; *P* < .001); for young adults, 8.8 (95% CI, 8.2-9.4) to 25.0 (95% CI, 23.5-26.6), increasing 16.2 (95% CI, 14.6-17.9; *P* < .001); for adults, 2.5 (95% CI, 2.3-2.7) to 7.4 (95% CI, 7.0-7.8), increasing 4.9 (95% CI, 4.4-5.3; *P* < .001) (Figure). States with the most restrictions had the highest increase post-*Dobbs*. Among adolescents, the pre-*Dobbs* rate was highest in states with parental consent and notification at 5.8 (95% CI, 5.1-6.5), vs 2.5 (95% CI, 2.2-2.7) in states without parental involvement, a difference of 3.4 (95% CI, 2.6-4.1; *P* < .001) (Table). Post-*Dobbs,* this gap widened to 11.2 (95% CI, 9.3-13.0; *P* < .001), with rates rising to 17.6 (95% CI, 15.8-19.4) in states with parental consent and notification, and 6.4 (95% CI, 6.0-6.9) in states without parental involvement.

**Figure.**
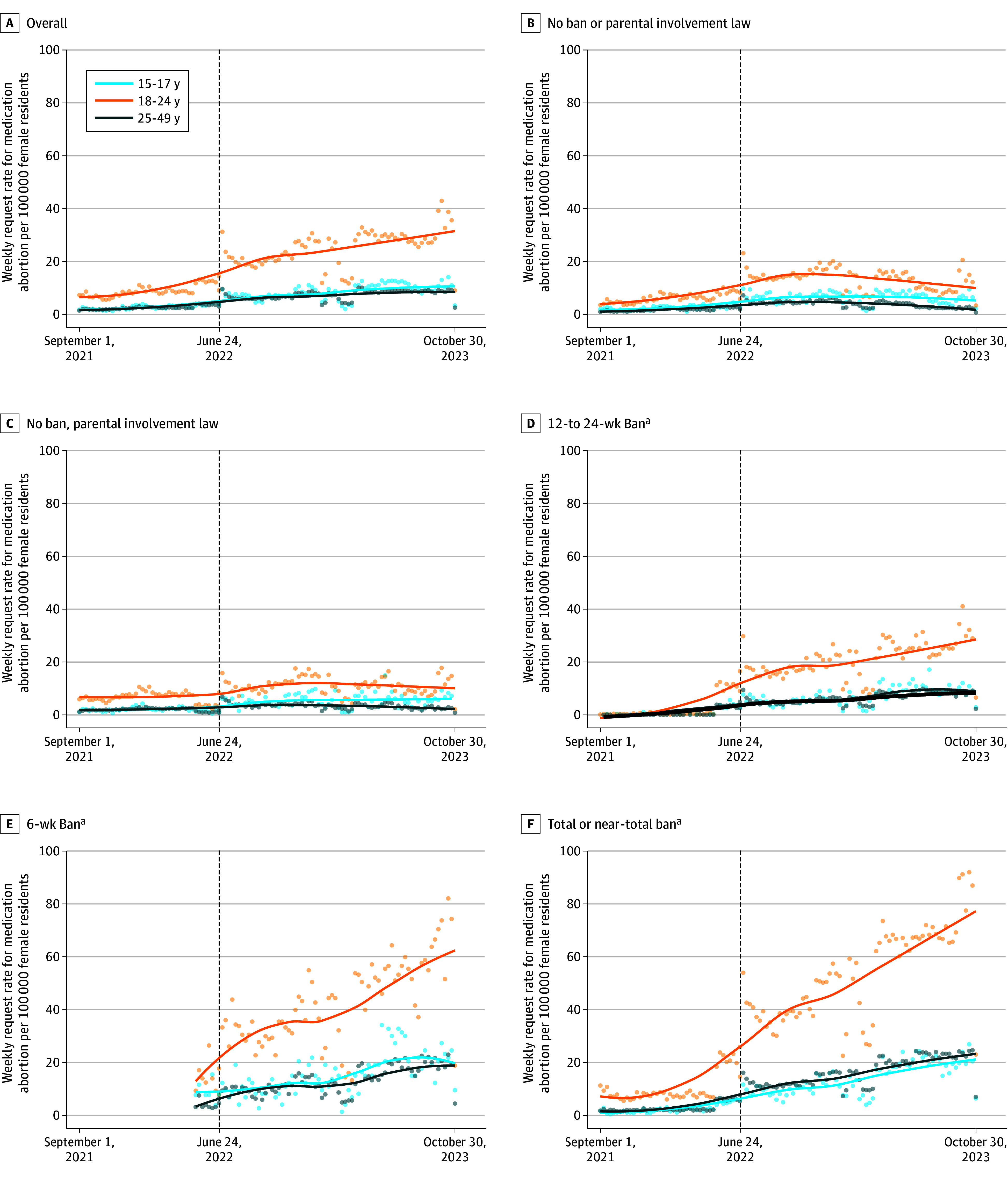
Weekly Request Rates for Medication Abortion via Telehealth Service by Age and Type of State-Level Abortion Policy Before and After the *Dobbs v Jackson Women’s Health* Decision The dashed line indicates the *Dobbs* decision. ^a^All states with bans have a parental involvement law.

**Table.** Weekly Request Rates for Medication Abortion via Telehealth Service Among Adolescents Aged 15 to 17 Years by Parental Involvement Law Before and After the *Dobbs v Jackson Women’s Health* Decision

Parental involvement law and time period	Mean weekly request rate per 100 000 female residents aged 15-17 y (95% CI)	Difference (95% CI)	*P* value
**Before *Dobbs* decision (September 1, 2021, to May 1, 2022)**			
None	2.5 (2.2 to 2.7)	0 [Reference]	
Notification	2.6 (2.2 to 3.0)	0.1 (−0.3 to 0.6)	.58
Consent	2.2 (1.9 to 2.4)	−0.3 (−0.7 to 0.0)	.09
Consent and notification	5.8 (5.1 to 6.5)	3.4 (2.6 to 4.1)	<.001
**After *Dobbs* decision (June 24, 2022, to October 30, 2023)**			
None	6.4 (6.0 to 6.9)	0 [Reference]	
Notification	8.3 (7.5 to 9.2)	1.9 (0.9 to 2.9)	<.001
Consent	8.1 (7.3 to 8.9)	1.7 (0.8 to 2.6)	<.001
Consent and notification	17.6 (15.8 to 19.4)	11.2 (9.3 to 13.0)	<.001

## Discussion

Prior research finds abortion restrictions are associated with increased demand for telehealth medication abortion.^6^ However, whether this trend extended to adolescents was previously unknown, a population facing the unique legal burden of mandatory parental involvement, in addition to gestation bans. We found that post-*Dobbs* increases in requests were highest among young adults, especially in states with restrictive abortion laws, and among adolescents, in states with gestational bans and parental consent and notification requirements. This study’s limitations included analysis of only one service, thus not representative of all people seeking medication abortion online. However, these findings highlight growing demand among adolescents and young adults in legally constrained environments. Young people appear to increasingly rely on online telemedicine services for abortion care, with compounding legal restrictions driving higher demand.

## Notes

Supplement 1. eMethods eReferences

Supplement 2. Data Sharing Statement
