# Supplementary material for: Adolescent and Young Adult Requests for Medication Abortion Through Online Telemedicine
Source: JAMA Health Forum. 2026 Feb 13;7(2):e256808. doi: 10.1001/jamahealthforum.2025.6808 (PMC12905652; doi:10.1001/jamahealthforum.2025.6808)
Supplement: Supplement 1. — eMethods eReferences [file jamahealthforum-e256808-s001.pdf]

Supplemental Online Content

Johnson DM, Starling JE, Gomperts R. Adolescent and young adult requests for medication abortion through online telemedicine. *JAMA Health Forum*. 2026;7(2):e256808. doi:10.1001/jamahealthforum.2025.6808

eMethods. Data Sources and Analysis
eReferences

This supplemental material has been provided by the authors to give readers additional information about their work.

© 2026 Johnson DM et al. JAMA Health Forum.

eMethods. Data Sources and Analysis

Data for this study were provided by an online telemedicine service that has provided medication abortion nationwide since 2018. As of July 2025, AbortionFinder.com and INeedAnA.com (the two most frequently updated online abortion search databases) report only a small number of telemedicine providers offering nationwide services without age restrictions. Prior research reports this service as a high-volume provider, receiving about 5000 medication requests each month. In contrast, another nationwide service reported serving 10 000 people in all of 2024.

Request rates were calculated per 100 000 female residents using state- and age-specific population estimates from the 2020 US Census API. For overall rates by age group, we created population denominators by summing the number of female residents in each age group across all states. For rates by age group and policy environment, we summed female residents in each age group within state groups classified by policy. The Guttmacher Institute provided policy categorizations.[1,2] Weekly request rates were then calculated by dividing the number of requests in each week by the relevant population denominator and multiplying by 100 000. This calculation was performed separately for the overall total and for each policy environment. (All states with a gestation ban and near or total ban also had an active parental involvement law.) Finally, we averaged weekly request rates over the pre-Dobbs (9/21/2021 to 6/23/2022) and post-Dobbs (6/24/2022 to 10/31/2023) periods.

We conducted sensitivity analysis to account for the Dobbs decision leak on 5/22/2025. In this analysis, we split the baseline period into two separate periods to account for the leak. This did not substantively change our findings.

© 2026 Johnson DM et al. JAMA Health Forum.

eReferences

1. Guttmacher Institute. Parental involvement in minor's abortions. Updated September 1, 2023. Accessed December 5, 2023. https://www.guttmacher.org/state-policy/explore/minors-access-abortion-care

2. Guttmacher Institute. Interactive map: US abortion policies and access after *Roe*. Accessed October 1, 2025. https://states.guttmacher.org/policies/

© 2026 Johnson DM et al. JAMA Health Forum.
