# Supplementary material for: Adolescent and Young Adult Requests for Medication Abortion Through Online Telemedicine
Source: JAMA Health Forum. 2026 Feb 13;7(2):e256808. doi: 10.1001/jamahealthforum.2025.6808 (PMC12905652; doi:10.1001/jamahealthforum.2025.6808)
Supplement: Supplement 2. — Data Sharing Statement [file jamahealthforum-e256808-s002.pdf]

Data Sharing Statement

Johnson. Adolescent and Young Adult Requests for Medication Abortion Through Online Telemedicine. *JAMA Health Forum*. Published February 13, 2026.
doi:10.1001/jamahealthforum.2025.6808

Data
Data available: No

Additional Information
Explanation for why data not available: Due to the sensitive nature of ordering medication abortion pills online in varying state legality contexts, at the request of Aid Access data will not be publicly available.
